# Energy requirements during weight loss and weight maintenance of two morbidly obese cats

**DOI:** 10.1186/1751-0147-57-S1-P3

**Published:** 2015-09-25

**Authors:** Ronald Jan Corbee

**Affiliations:** 1 Department of Clinical Science, Utrecht University, Utrecht, Netherlands

## Introduction

Two morbidly obese cats were presented at the weight management clinic. The first cat was a domestic shorthair with a body weight of 10.82kg. The second cat was a domestic shorthair with a body weight of 13.42kg. At presentation, both cats suffered from obstipation, and dull hair coat.

## Objectives

To achieve weight loss to target weight and to maintain target weight for at least 2 years and to evaluate energy requirements during weight loss in morbidly obese cats.

## Methods

The two cats were put on a weight reduction diet with high protein (34% on dry matter basis) and high fiber (14.1% on dry matter basis) content at 0.8xRER (resting energy requirement). The amount of food was adjusted according to the progress of weight loss, monitored by 2-weekly body weight measurements. The amount of food eaten and recommended were recorded.

## Results

Both cats reached their target weight of 3.8kg and 4.5kg, respectively. Both cats maintained their target weight for two years with minor adaptions of food intake during weight loss and weight maintenance phase. Constipation and dull hair coat resolved quickly.

## Conclusions

Weight loss by reducing food intake and by the use of a high protein, high fiber diet are very effective, even in morbidly obese cats. Client communication and proper follow up are key factors for successful obesity management. Metabolic adaptations occur during weight loss and weight maintenance, resulting in altered enery requirents, which therefore requires lifelong follow up and adaptations in food intake.

